# Effect of diet-induced weight loss on lipoprotein(a) levels in obese individuals with and without type 2 diabetes

**DOI:** 10.1007/s00125-017-4246-y

**Published:** 2017-04-06

**Authors:** Kirsten A. Berk, Reyhana Yahya, Adrie J. M. Verhoeven, Jeanette Touw, Frank P. Leijten, Elisabeth F. van Rossum, Vincent L. Wester, Mirjam A. Lips, Hanno Pijl, Reinier Timman, Gertraud Erhart, Florian Kronenberg, Jeanine E. Roeters van Lennep, Eric J. G. Sijbrands, Monique T. Mulder

**Affiliations:** 1 Department of Internal Medicine, Erasmus Medical Center, Office Ee800, PO Box 2040, 3000 CA Rotterdam, the Netherlands; 2 Department of Internal Medicine, Leiden University Medical Center, Leiden, the Netherlands; 3 Department of Psychiatry, Section of Medical Psychology and Psychotherapy, Erasmus Medical Center, Rotterdam, the Netherlands; 4 Division of Genetic Epidemiology, Department of Medical Genetics, Molecular and Clinical Pharmacology, Medical University of Innsbruck, Innsbruck, Austria

**Keywords:** Apolipoprotein(a), Bariatric surgery, Diet, Lipoprotein(a), Obesity, Type 2 diabetes, Weight loss

## Abstract

**Aims/hypothesis:**

Elevated levels of lipoprotein(a) [Lp(a)] are an independent risk factor for cardiovascular disease (CVD), particularly in individuals with type 2 diabetes. Although weight loss improves conventional risk factors for CVD in type 2 diabetes, the effects on Lp(a) are unknown and may influence the long-term outcome of CVD after diet-induced weight loss. The aim of this clinical study was to determine the effect of diet-induced weight loss on Lp(a) levels in obese individuals with type 2 diabetes.

**Methods:**

Plasma Lp(a) levels were determined by immunoturbidimetry in plasma obtained before and after 3–4 months of an energy-restricted diet in four independent study cohorts. The primary cohort consisted of 131 predominantly obese patients with type 2 diabetes (cohort 1), all participants of the Prevention Of Weight Regain in diabetes type 2 (POWER) trial. The secondary cohorts consisted of 30 obese patients with type 2 diabetes (cohort 2), 37 obese individuals without type 2 diabetes (cohort 3) and 26 obese individuals without type 2 diabetes who underwent bariatric surgery (cohort 4).

**Results:**

In the primary cohort, the energy-restricted diet resulted in a weight loss of 9.9% (95% CI 8.9, 10.8) and improved conventional CVD risk factors such as LDL-cholesterol levels. Lp(a) levels increased by 14.8 nmol/l (95% CI 10.2, 20.6). In univariate analysis, the change in Lp(a) correlated with baseline Lp(a) levels (*r* = 0.38, *p* < 0.001) and change in LDL-cholesterol (*r* = 0.19, *p* = 0.033). In cohorts 2 and 3, the weight loss of 8.5% (95% CI 6.5, 10.6) and 6.5% (95% CI 5.7, 7.2) was accompanied by a median increase in Lp(a) of 13.5 nmol/l (95% CI 2.3, 30.0) and 11.9 nmol/l (95% CI 5.7, 19.0), respectively (all *p* < 0.05). When cohorts 1–3 were combined, the diet-induced increase in Lp(a) correlated with weight loss (*r* = 0.178, *p* = 0.012). In cohort 4, no significant change in Lp(a) was found (−7.0 nmol/l; 95% CI -18.8, 5.3) despite considerable weight loss (14.0%; 95% CI 12.2, 15.7).

**Conclusions/interpretation:**

Diet-induced weight loss was accompanied by an increase in Lp(a) levels in obese individuals with and without type 2 diabetes while conventional CVD risk factors for CVD improved. This increase in Lp(a) levels may potentially antagonise the beneficial cardiometabolic effects of diet-induced weight reduction.

## Introduction

Cardiovascular disease (CVD) is the main cause of morbidity and mortality in obese individuals with and without type 2 diabetes [1–3]. The risk of CVD in obese patients with type 2 diabetes has been attributed to age, smoking, hyperglycaemia, hypertension and dyslipidaemia [2]. Weight loss via lifestyle programmes, consisting of diet and physical activity, results in an improvement in conventional CVD risk factors and is first-line therapy to slow down the development of type 2 diabetes and the progression of its complications in overweight or obese individuals [4, 5].

Lipoprotein a [Lp(a)] is an independent risk factor for CVD [6–12]. Lp(a) consists of an LDL-like particle with an additional apolipoprotein (a) [Apo(a)] attached to it. Plasma Lp(a) concentrations vary highly between individuals and are largely genetically determined by the number of copies of kringle-IV type 2 (KIV-2) in the Apo(a) protein [Apo(a) isoform] [13–16]. A low number of copies of KIV-2, associated with elevated levels of Lp(a), has been shown to increase the risk of CVD [17]. A recent prospective population-based cohort of 56,367 participants showed a significantly higher contribution of Lp(a) levels to CVD and risk of myocardial infarction in patients with type 2 diabetes than in control participants without type 2 diabetes [18]. About 25% of the variance in Lp(a) levels has been attributed to lifestyle [19]. Weight loss in obese individuals has been reported to affect Lp(a) levels, but the results are controversial [20–23]. The effect of weight loss on plasma Lp(a) levels in type 2 diabetes has not yet been determined.

The aim of the current study was to determine the effect of diet-induced weight loss on Lp(a) levels in obese patients with type 2 diabetes. In order to confirm our findings we also examined the effect of weight loss on Lp(a) levels in three independent cohorts of obese patients with or without type 2 diabetes. As a secondary aim, we assessed the influence of Apo(a) isoforms on diet-induced changes in Lp(a) level in individuals with type 2 diabetes.

## Methods

### Participants and interventions

The effect of weight loss was examined in four independent cohorts. The primary cohort (cohort 1, *n* = 131) consisted of overweight and obese individuals (BMI >27 kg/m^2^, 93% obese) with type 2 diabetes who participated in the run-in phase of the Prevention Of Weight Regain (POWER) trial (trial registration no. NTR2264) [24]. This trial aimed to study long-term weight maintenance after the run-in diet phase. The sample size of 131 patients was sufficient to find a difference of 10.6 nmol/l (5 mg/dl) in Lp(a) level with a baseline-to-end correlation of 0.95 between the measurements, an α of 0.05 and a power of 0.90. The diet started with 8 weeks of a diet very low in energy (very low calorie diet [VLCD]) of approximately 3000 kJ (750 kcal) per day, consisting of two meal replacements (Glucerna, Abbott Nutrition, Lake Forest, IL, USA) and a small dinner daily. Thereafter, energy intake was slowly increased up to approximately 5500 kJ (1300 kcal) per day (a low-energy diet) over 12 weeks. Some of the baseline characteristics and effect of the diet on body weight in cohort 1 have previously been reported [25].

Cohort 2 (*n* = 30) also consisted of overweight and obese patients (80% obese) with type 2 diabetes, who were recruited after the POWER trial had finished, to study the implementation of a VLCD for weight loss in type 2 diabetes. The participants underwent the same dietary intervention as the patients in the primary cohort. Cohorts 1 and 2 were both recruited from the outpatient diabetes clinic of the Erasmus Medical Center, Rotterdam, the Netherlands. To reduce risk of hypoglycaemia, doses of insulin and sulfonylurea derivates were lowered before the start of the diet but after baseline measurements had been made. During the diet, the insulin dose was regularly adjusted to achieve optimal glycaemic control. Metformin use was continued. Only two participants were taking glucagon-like peptide 1 (GLP-1) receptor agonist treatment, which was continued during the intervention period. Statin treatment was kept unchanged during the study.

Cohort 3 consisted of 37 obese individuals without type 2 diabetes, who were recruited at the Obesity Center ‘Centrum voor Gezond Gewicht’ of the Erasmus Medical Center. They underwent a 3 month dietary intervention consisting of a 2000 kJ (500 kcal) per day reduction in intake relative to baseline (low-energy diet), with macronutrient and micronutrient content in line with national dietary guidelines, while exercise was encouraged.

Cohort 4 consisted of 26 obese individuals without type 2 diabetes who underwent gastric banding (*n* = 10) or a gastric bypass procedure (*n* = 16). These participants were recruited at the Leiden University Medical Center, Leiden, the Netherlands. No specific diet was recommended beyond a staged meal progression during the first 3 months after surgery. Analyses were performed at baseline and 3 months after surgery.

The dietary intervention studies and Lp(a) analysis of previously collected clinical samples were approved by the Medical Ethics Committee of the Erasmus Medical Center (reference numbers MEC-2009-143, MEC-2014-090 and MEC 2016-604). The bariatric surgery study and use of the samples was approved by the Medical Ethics Committee of the Leiden University Medical Center (reference number MEC P08.215). All investigations were carried out in accordance with the principles of the Declaration of Helsinki (2008). All participants provided written informed consent.

### Measurements

Blood samples were obtained after an overnight fast and were stored at −80°C until further analysis. Demographic variables were recorded, and weight, height and waist circumference (except for cohort 4) were measured. Ethnicity was expressed as white or non-white. HbA_1c_, fasting glucose, total cholesterol, LDL-cholesterol, HDL-cholesterol and triacylglycerol were measured using standard laboratory techniques.

### Lp(a) measurement

Plasma Lp(a) concentrations were measured using a particle-enhanced immunoturbidimetric assay, which was largely independent of Apo(a) KIV repeat number (Diagnostic System #171399910930; DiaSys Diagnostic System, GmbH, Holzheim, Germany) [26]. Plasma samples were stored at −80°C for 0.5–5 years and thawed for the first time prior to this analysis. For each individual, levels at baseline and after intervention were measured in the same run. The detection limit of the assay was 6 nmol/l, and the mean intra-assay variability was 2.8%. Interference of triacylglycerol with Lp(a) measurements was minimal, as measured Lp(a) levels were less than 5% affected by the addition of plasma containing different concentrations of triacylglycerol (ranging from 0 to 12 mmol/l) to plasma with a relatively high Lp(a) concentration (169 or 338 nmol/l). Repeated sampling in 27 healthy control individuals at an interval of 2–6 months did not reveal significant differences in median Lp(a): 29.3 nmol/l (interquartile range [IQR] 17.5–87.8) vs 26.4 nmol/l (IQR 12.4–60.3), *p* = 0.087, for day 0 and after 2–6 months, respectively.

In the primary cohort (cohort 1), the Apo(a) KIV repeat number was determined by immunoblotting, as previously described [27, 28]. When two distinct Apo(a) isoforms were present, the band representing the smaller isoform showed the strongest intensity in most cases and was used as a continuous variable. Apo(a) KIV repeat numbers were stratified in two groups as previously described [28]: low molecular weight (mass) (LMW) when at least one isoform with 22 or fewer KIV repeats was present, and high molecular weight (mass) (HMW) when only isoforms with more than 22 repeats were present.

### Statistical analysis

Normality of the data and homogeneity of variances were tested using the Shapiro–Wilk test and Levene’s test. Variables were expressed as mean ± SD or as median with IQR, and were tested for statistical significance using a two-sided paired-sample *t* test or a Wilcoxon ranking test, depending on the normality of the data. Medians and 95% CIs were calculated using ratio statistics, and median differences were analysed using a related-samples Hodges–Lehman test. Owing to the low numbers in cohorts 2, 3 and 4, in-depth analyses were performed only for cohort 1. We determined Spearman correlations of both baseline Lp(a) levels and change in Lp(a) with different variables of weight loss and glycaemic control.

Mann–Whitney U tests were used to analyse the difference in baseline Lp(a) levels between the LMW and HMW subgroups. Repeated-measurements multivariate ANOVA (MANOVA) analysis (on Blom-transformed outcome variables) was used to analyse the difference in change in Lp(a) between subgroups. SPSS version 21.0 (IBM, Armonk, NY, USA) and GraphPad Prism version 5 (GraphPad Software, La Jolla, CA, USA) were used for the statistical analyses.

## Results

### Effect of diet on obese patients with type 2 diabetes (cohort 1)

The characteristics of the primary cohort (cohort 1) at baseline and after intervention are shown in Table 1. The 131 individuals were predominantly obese, as 93% had a BMI greater than 30 kg/m^2^. The remainder had a BMI >27 and ≤30 kg/m^2^. This cohort had a mixed ethnic background (56% white, and 44% non-white: South-Asian and African). Baseline Lp(a) levels correlated negatively with Apo(a) KIV repeat number (*r* = −0.53, *p* < 0.001), baseline weight (*r* = −0.18, *p* = 0.046), HbA_1c_ (*r* = −0.20, *p* = 0.022), fasting triacylglycerol (*r* = −0.19, *p* = 0.032) and ethnicity (*r* = −0.34, *p* < 0.001), and positively with LDL-cholesterol (*r* = 0.18, *p* = 0.038). We found no correlation of baseline Lp(a) levels with sex (*r* = 0.08, *p* = 0.389), fasting glucose (*r* = −0.17, *p* = 0.057) or fasting insulin levels (*r* = −0.06, *p* = 0.494). Participants of white origin had lower baseline Lp(a) levels than non-white participants: median 25.7 nmol/l (IQR 5.7–120.1) vs 122.0 nmol/l (IQR 34.0–214.6) (*p* < 0.001).

**Table 1:** Characteristics of the study cohorts before and after intervention

Variables	Cohort 1 (*n* = 131)		Cohort 2 (*n* = 30)		Cohort 3 (*n* = 37)		Cohort 4 (*n* = 26)	
	Before	After	Before	After	Before	After	Before	After
Age, years (range)	54 (26–74)	–	55 (34–70)	–	42 (18–63)	–	48 (34–59)	–
Sex, n (%) female	75 (57%)	–	15 (50%)	–	29 (78%)	–	26 (100%)	–
Years after diagnosis of T2D	10.0 (3.0–15.0)	–	5.0 (2.0–10.0)	–	–	–	–	–
Weight (kg)	105.0 ± 19.1	94.5 ± 17.3^***^	103.2 ± 23.3	94.2 ± 21.7^***^	111.4 ± 17.1	104.3 ± 16.7^***^	124.0 ± 11.8	106.6 ± 11.2^***^
BMI (kg/m^2^)	36.8 ± 5.6	33.1 ± 5.2^***^	34.8 ± 6.6	31.8 ± 6.6^***^	38.4 ± 4.7	35.9 ± 4.5^***^	43.7 ± 3.2	37.4 ± 3.5^***^
Waist circumference (cm)	119.8 ± 12.9	110.8 ± 11.9^***^	113.1 ± 12.3	106.0 ± 12.3^**^	106.2 ± 15.1	98.3 ± 13.8^***^	–	–
HbA_1c_ (%)	7.7 (6.9–8.6)	7.0 (6.1–8.2)^***^	7.5 (7.0–8.2)	6.6 (6.0–8.2)^**^	5.5 (5.3–5.8)	5.4 (5.2–5.7)^**^		
HbA_1c_ (mmol/mol)	61.0 (52.0–71.0)	53.0 (43.0–66.0)^***^	58.0 (53.0–65.8)	49.0 (42.0–66.0)^**^	37.0 (34.0–39.5)	36.0 (33.0–38.5)^**^	–	–
Fasting glucose (mmol/l)	8.8 (6.9–10.8)	7.3 (6.1–9.3)^***^	8.7 (7.0–10.5)	7.4 (6.5–9.3)	5.3 (5.0–5.8)	5.1 (4.8–5.4)^**^	5.1 (4.7–5.2)	4.9 (4.4–5.3)
Total cholesterol (mmol/l)	4.4 (3.7–5.1)	4.1 (3.5–4.8)^***^	3.9 (3.6–5.1)	4.2 (3.5–5.5)	5.2 (4.3–5.7)	4.6 (4.1–5.2)^***^	4.7 (3.8–5.8)	4.0 (3.5–4.9)^**^
LDL-cholesterol (mmol/l)	2.5 (2.1–3.1)	2.4 (1.8–2.9)^***^	2.4 (2.0–3.2)	2.2 (1.7–3.3)	3.4 (3.0–4.0)	3.1 (2.7–3.6)^***^	2.8 (2.3–3.6)	2.3 (1.8–3.0)^**^
HDL-cholesterol (mmol/l)	1.1 (1.0–1.3)	1.2 (1.0–1.4)^**^	1.2 (1.0–1.5)	1.2 (1.1–1.5)	1.3 (1.1–1.4)	1.2 (1.1–1.4)^**^	1.1 (1.0–1.3)	1.0 (0.9–1.2)^**^
Triacylglycerol (mmol/l)	1.8 (1.2–2.6)	1.4 (1.0–2.0)^***^	1.5 (1.1–2.5)	1.4 (0.9–2.0)	1.3 (1.0–1.8)	1.1 (0.9–1.4)^*^	1.2 (0.9–1.8)	1.2 (1.0–1.4)
Lp(a) (nmol/l)	40.9 (13.9–159.5)	55.9 (23.0–201.1)^***^	56.9 (12.4–148.9)	61.5 (20.4–185.9)^*^	27.0 (2.1–75.2)	45.2 (22.7–94.5)^**^	36.4 (17.2–91.5)	20.6 (6.3–104.1)

Data are mean ± SD, or median (IQR)

*p < 0.05, **p < 0.01, ***p < 0.001; difference before–after intervention

T2D, type 2 diabetes

The diet resulted in a weight loss of 10.2 kg (95% CI 9.2, 11.3), which was equivalent to 9.9% (95% CI 8.9, 10.8) of initial body weight. Both BMI and waist circumference decreased significantly (*p* < 0.001 for all). HbA_1c_ and fasting glucose levels decreased (*p* < 0.001 for both), indicating improved glycaemic control. Lipid variables also improved during the dietary intervention (*p* < 0.01 for all, Table 1).

**Fig. 1:**
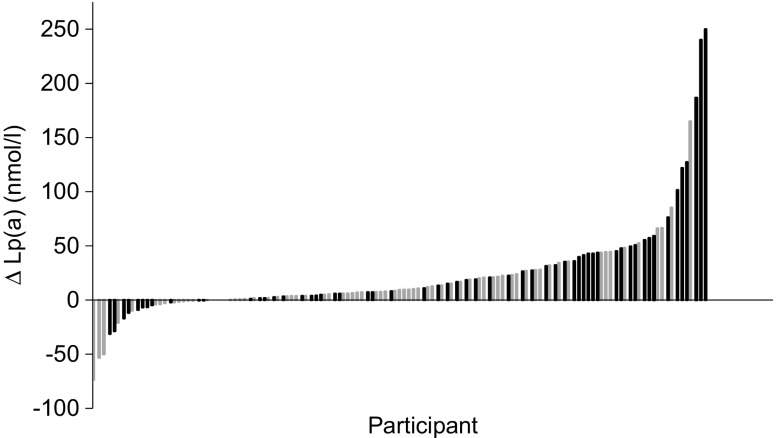
Diet-induced changes in Lp(a) level per individual in cohort 1 (*n* = 131). Individual participants (*x*-axis) are arranged according to the diet-induced change in Lp(a) level. Grey bars, white participants; black bars, non-white participants

Lp(a) levels increased significantly from 40.9 nmol/l (IQR 13.9–159.5) to 55.9 nmol/l (IQR 23.0–201.1) (*p* < 0.001, Table 1). Figure 1 shows a waterfall plot of the changes in Lp(a) per individual. Of the 131 participants, 49 showed an increase of over 21 nmol/l (10 mg/dl), and only six showed a decrease of 21 nmol/l or more (10 mg/dl). The median increase in Lp(a) levels in cohort 1 was 14.8 nmol/l (95% CI 10.2, 20.6).

The change in Lp(a) correlated with baseline Lp(a) levels (*r* = 0.38, *p* < 0.001) and with the change in fasting glucose (*r* = −0.17, *p* = 0.049) and LDL-cholesterol (*r* = 0.19, *p* = 0.033). The correlations with change in fasting glucose and LDL-cholesterol disappeared after correction for baseline Lp(a) levels. The change in Lp(a) did not correlate with sex (*r* = −0.04, *p* = 0.543) or change in weight (*r* = −0.14, *p* = 0.116). The change in Lp(a) also correlated with ethnicity (white vs non-white: *r* = −0.17, *p* = 0.048), although this did not happen after correction for baseline Lp(a) levels. The Lp(a) response to the diet did not differ between white and non-white individuals in a repeated-measurements MANOVA (F_(1;129)_ = 0.199, *p* = 0.656). In cohort 1, 95 out of the 131 (73%) participants used statins; the diet-induced change in Lp(a) levels was similar whether or not statins were used (F_(1;129)_ = 0.669, *p* = 0.415).

Excluding two possible outliers with an increase Lp(a) level of ≥211 nmol/l, the outcome was similar.

### Effect of Apo(a) isoform on diet-induced changes in Lp(a) levels in cohort 1

**Fig. 2:**
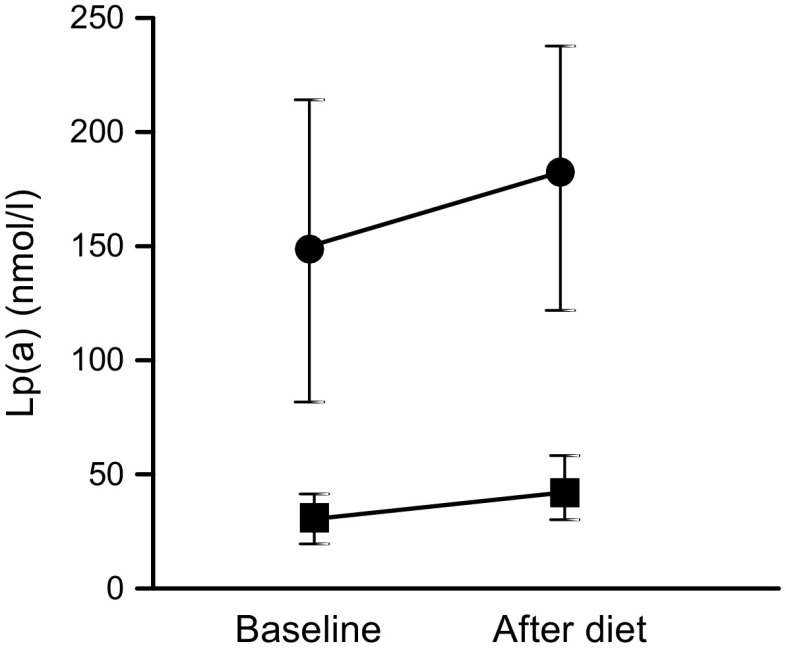
The effect of the dietary intervention on Lp(a) level in the Apo(a) isoform subgroups in cohort 1. Medians and 95% CIs of Lp(a) levels before and after the dietary intervention for the LMW Apo(a) isoform group (circles, *n* = 43) and the HMW Apo(a) isoform group (squares, *n* = 88)

Forty-three participants had an LMW and 88 an HMW Apo(a) isoform. As expected, baseline Lp(a) levels were significantly higher in the LMW than the HMW subgroup (148.8 nmol/l [IQR 26.6–297.9] vs 30.6 nmol/l [IQR 6.5–119.4]; *p* < 0.001). Lp(a) levels increased during the dietary intervention to 182.7 nmol/l (IQR 37.3–327.5; *p* < 0.001) in the LMW subgroup and to 41.6 nmol/l (IQR 15.4–139.9; *p* < 0.001) in the HMW subgroup (Fig. 2). The diet-induced effect on Lp(a) did not significantly differ between the LMW and the HMW subgroup (F_(1;129)_ = 1.68, *p* = 0.197). The alteration in Lp(a) levels correlated strongly with baseline Lp(a) level in the HMW subgroup (*r* = 0.43, *p* < 0.001) but not in the LMW subgroup (*r* = 0.24, *p* = 0.118).

### Long-term effect

Of the 131 participants in cohort 1, 69 consented to provide an additional blood sample 20 months after finishing the dietary intervention. This subgroup was older (55.6 vs 51.8 years, *p* = 0.016), had a longer history of type 2 diabetes (12.2 vs 8.8 years, *p* = 0.017) and had lost more weight during the intervention (12.1 vs 8.6 kg, *p* = 0.001), but did not differ from the other participants in sex distribution, ethnicity, baseline Lp(a), BMI, HbA_1c_ and LDL-cholesterol, nor in change in Lp(a) during the diet. In this subgroup, Lp(a) levels increased from 40.9 nmol/l (IQR 15.6–151.7) to 55.1 nmol/l (IQR 24.7–200.2) during the dietary intervention. Twenty months after the diet, patients had regained an average of 6.8 ± 5.5 kg of body weight but were still 5.2 ± 6.0 kg below baseline weight. Lp(a) levels decreased to 43.9 nmol/l (IQR 12.2–157.8), which was no longer statistically different from baseline levels (*p* = 0.050). Weight regain was not correlated with the decrease in Lp(a) levels from the end of the intervention to 20 months after the intervention (*r* = −0.06, *p* = 0.626).

### Effect of weight loss on Lp(a) levels in secondary cohorts

The characteristics of the cohorts 2–4 at baseline and after the intervention are shown in Table 1. Cohort 2, consisting predominantly of obese patients with type 2 diabetes, showed effects of the diet similar to the primary cohort. Weight loss was 9.0 kg (95% CI 6.7, 11.3), or 8.5% (95% CI 6.5, 10.6) of initial body weight, and both BMI and waist circumference decreased significantly (*p* < 0.01 for all). HbA_1c_ level also decreased (*p* = 0.001), but changes in fasting glucose and lipid variables (total cholesterol, triacylglycerol, LDL-cholesterol and HDL-cholesterol) did not reach statistical significance in this small group (Table 1). During dieting, Lp(a) increased from 56.9 nmol/l (IQR 12.4–148.9) to 61.5 nmol/l (IQR 20.4–185.9) (*p* = 0.018; Table 1). The median increase in Lp(a) was 13.5 nmol/l (95% CI 2.3, 30.0).

In cohort 3, which consisted of obese individuals without type 2 diabetes, the dietary intervention led to a weight loss of 7.1 kg (95% CI 6.3, 8.0), or 6.5% (95% CI 5.7, 7.2) of initial body weight, and to significant reductions in BMI and waist circumference (*p* < 0.001 for all). Although these participants did not have type 2 diabetes, HbA_1c_ and fasting glucose levels improved in this group (*p* = 0.002 and *p* = 0.003, respectively). In addition, lipid variables improved significantly (*p* < 0.05 for all). Lp(a) levels increased from 27.0 nmol/l (IQR 2.1–75.2) to 45.2 nmol/l (IQR 22.7–94.5) (*p* = 0.001; Table 1). The median increase in Lp(a) was 11.9 nmol/l (95% CI 5.7, 19.0).

Cohort 4 consisted of obese individuals without type 2 diabetes who underwent bariatric surgery and were followed up for 3 months. This intervention resulted in a weight loss of 17.4 kg (95% CI 15.0, 19.8), or 14.0% (95% CI 12.2, 15.7) of initial body weight (*p* < 0.001). During this period, most lipid variables improved significantly (Table 1). Lp(a) levels were lower after the intervention than before (falling from 36.4 nmol/l [IQR 17.2–91.5] to 20.6 nmol/l [IQR 6.3–104.1]), but this result did not reach statistical significance in this small group (Table 1). The median difference in Lp(a) level was −7.0 nmol/l (95% CI −18.8, 5.3).

**Fig. 3:**
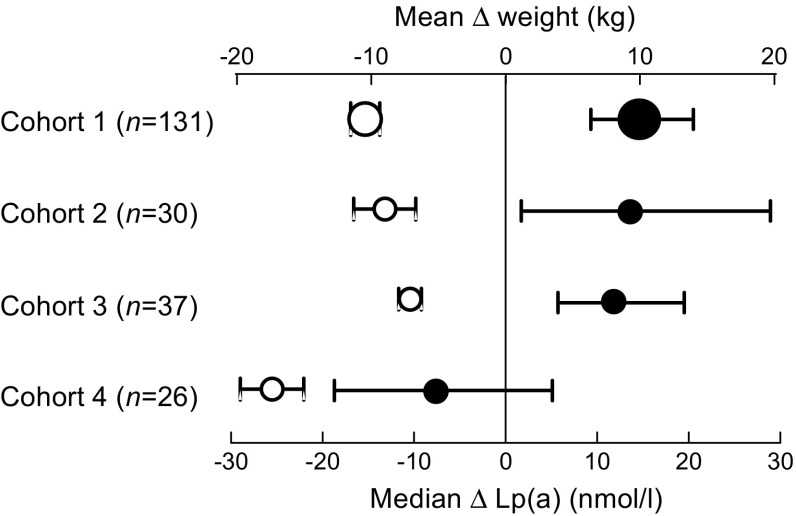
ΔLp(a) and Δweight in the four independent study cohorts. Means and 95% CI for Δweight (white circles) and medians with 95% CIs for ΔLp(a) (black circles) in the four cohorts. The size of the symbols reflects the number of participants

Figure 3 summarises the results obtained for the four independent cohorts. The relationship between weight loss and increase in Lp(a) levels was similar for the first three cohorts. When cohorts 1–3 were considered together, the increase in Lp(a) correlated with the diet-induced weight loss (*n* = 198, *r* = −0.18, *p* = 0.012). This relationship was not observed for cohort 4, which consisted of individuals who lost weight after bariatric surgery.

## Discussion

Our data show that diet-induced weight loss increased Lp(a) levels in overweight and obese individuals irrespective of the presence or absence of type 2 diabetes. Repeated sampling in healthy control participants at an interval of 2–6 months showed that the increase in Lp(a) levels was not explained by general environmental changes over time or by assay artefacts. In patients with type 2 diabetes, the extent of the increase in Lp(a) was mainly determined by baseline Lp(a) level, with the highest increase seen in individuals with the highest baseline levels. This effect on Lp(a) was independent of the Apo(a) isoform. Such an increase in Lp(a) levels was not observed in individuals who underwent bariatric surgery, suggesting that weight loss per se does not increase Lp(a) levels.

Previous studies have not shown a change in Lp(a) levels in obese adults after various dietary interventions aimed at weight loss [21–23]. In these studies, weight-reducing drugs and diets different from ours were tested, and patients with type 2 diabetes were not included. One study reported a decrease in Lp(a) levels in obese children [20]. This discrepancy in relation to our study may be explained by different age-related hormonal states or by differences in dietary composition. The type and content of fat in the diet may be an important determinant of the dietary effect on Lp(a) levels. An increased intake of total and saturated fat has been found to decrease Lp(a) levels, while an increased intake of monounsaturated fatty acids tended to increase Lp(a) levels in healthy individuals and those with metabolic dysregulation [29–31]. Faghihnia et al [30] have suggested that a low-fat diet results in an increase in Lp(a) levels that may be due to an altered metabolism of Lp(a) particles. The dietary interventions used in our cohorts 1–3 were all based on a low intake of total and saturated fat, while no specific dietary restrictions were prescribed for the participants in the cohort who underwent bariatric surgery. We previously reported that, in a random subset of participants in cohort 1, our dietary intervention lowered plasma levels of the soluble form of the LDL receptor relative with 11 ligand-binding repeats (sLR11) [32]. However, diet-induced changes in sLR11 and Lp(a) levels did not correlate with each other (*r* = −0.07, *p* = 0.635). In participants in cohort 1 from whom blood samples were available at 20 months of follow-up, Lp(a) levels had almost returned to baseline values, whereas the initial weight reduction was only partially reversed by weight regain. This suggests that the increase in Lp(a) levels was an acute effect of the diet. Unfortunately, we do not have information about the diet during the prolonged follow-up. Future studies on the effect on Lp(a) of weight-reducing diets with different fat contents in obese patients with and without type 2 diabetes are warranted.

High Lp(a) levels have consistently been associated with an increased risk of coronary heart disease [6, 9], and results from genetic studies indicate a causal association between high Lp(a) levels and CVD [17, 33, 34]. The risk of CVD associated with high Lp(a) levels is notably higher in individuals with than without type 2 diabetes [18]. The dose–response relationship of Lp(a) levels with CVD risk has been shown to be curvilinear in shape, with no evidence of a threshold [35]. This suggests that the increase in Lp(a) levels induced by weight loss dieting observed in our study might increase the risk of CVD. This could potentially reduce the beneficial cardiometabolic effects that result from the improvement in conventional CVD risk factors after diet-induced weight loss. In the Look AHEAD (Action for Health in Diabetes) study (NCT00017953), the incidence of CVD was not reduced by a low-energy, low-fat diet and physical activity in patients with type 2 diabetes after 10 years of follow-up, despite an improvement in conventional risk factors for CVD [5]. Hypothetically, a parallel increase in Lp(a) levels could be one of the explanations why CVD events were not reduced by this lifestyle change. However, effects on Lp(a) levels were not reported in the Look AHEAD trial. Randomised clinical trials addressing the effect of alterations in Lp(a) levels with lifestyle changes or medication on hard clinical endpoints or CVD risk are needed. Recently, the short-term efficacy and safety of two specific Lp(a)-lowering agents has been reported [36]. Long-term effects on cardiovascular endpoints are awaited.

In participants who underwent bariatric surgery, weight loss was not accompanied by an increase in Lp(a) level. Two previous studies have shown that bariatric surgery-induced weight loss in obese individuals was accompanied by a decrease in Lp(a) levels [37, 38], whereas another study found no significant effect [39]. The effects of bariatric surgery on bile acid flow and signalling, inflammation, release of gastrointestinal hormones, the gut microbiome and the wound healing processes may all have had an impact on Lp(a), resulting in the absence of a weight loss-induced increase in Lp(a) levels [40–44].

The baseline Lp(a) levels in our two cohorts with type 2 diabetes (cohorts 1 and 2) were relatively high compared with the two cohorts without type 2 diabetes (cohorts 3 and 4), whereas in the Women’s Health Study and Copenhagen City Heart Study the Lp(a) levels in participants with diabetes were significantly lower than the Lp(a) levels in the control participants [45, 46]. Non-white individuals, in particular those of South-Asian ancestry, display markedly higher Lp(a) levels than white individuals [47–49], and are over-represented in our cohorts with type 2 diabetes. The change in Lp(a) during the diet was correlated with ethnicity. However, in the repeated-measurements analysis we found no difference between the white and non-white populations in ΔLp(a). This suggests that non-white individuals have higher baseline Lp(a) levels, and therefore show the highest absolute change in Lp(a) levels on dieting, but that the relative change is similar to that in white individuals.

The strengths of this study are its prospective design and the use of four independent cohorts for investigating the effect of weight loss on Lp(a), which more than doubled the total number of participants who have so far been studied in relation to this topic. Our study is descriptive in nature. Future studies should clarify the mechanisms underlying the increase in Lp(a) levels on diet-induced weight loss, as well as the consequence of weight loss for the functionality of Lp(a). As all the participants had been referred to a tertiary centre, our findings may not be applicable to the entire population of overweight and obese patients with or without type 2 diabetes. We found that the effect of diet-induced weight loss on Lp(a) levels occurred irrespective of the presence or absence of type 2 diabetes. However, some of the individuals in cohorts 3 and 4 may have had impaired glucose tolerance, since the classification was based on fasting glucose level and not on an oral glucose tolerance test. Finally, a long-term follow-up study is required to determine whether elevated Lp(a) levels after weight loss dieting affect the incidence of CVD in obese patients with and without type 2 diabetes.

In conclusion, Lp(a) levels increased significantly in obese individuals with and without type 2 diabetes during diet-induced weight loss, but not in individuals who underwent bariatric surgery. This may hypothetically reduce the beneficial cardiometabolic effects of a diet-induced weight loss. Therefore, Lp(a) may be an additional target in overweight and obese individuals on a energy-restricted diet to reduce the risk of CVD. Long-term follow-up studies are required to establish whether adding a specific Lp(a)-lowering agent to a dietary intervention will improve long-term CVD outcome in obese individuals with and without type 2 diabetes.

## Abbreviations

AHEAD: Action for Health in Diabetes
Apo(a): Apolipoprotein (a)
CVD: Cardiovascular disease
HMW: High molecular weight
IQR: Interquartile range
KIV: Kringle-IV type 2
LMW: Low molecular weight
Lp(a): Lipoprotein (a)
MANOVA: Multivariate ANOVA
sLR11: Soluble form of the LDL receptor relative with 11 ligand-binding repeats
VLCD: Very low calorie diet
